# Lower breathing frequencies in personalized slow-paced breathing enhance relaxation and reduce arousal

**DOI:** 10.1016/j.isci.2026.115803

**Published:** 2026-04-17

**Authors:** Lukas Moebus, Manuel Spitschan, Felix Ehrlenspiel

**Affiliations:** 1 Department of Health and Sport Sciences, TUM School of Medicine and Health, Technical University of Munich, 80809 Munich, Germany; 2 Institute for Advanced Study (TUM-IAS), Technical University of Munich, 85748 Garching, Germany; 3 Max Planck Research Group Translational Sensory & Circadian Neuroscience, Max Planck Institute for Biological Cybernetics, 72076 Tübingen, Germany

**Keywords:** health sciences, psychiatry, physiology, psychology

## Abstract

Among all vital functions, breathing is uniquely under both autonomic and volitional control. This dual control enables conscious recognition and regulation of arousal. While breathwork affects arousal and relaxation, research rarely investigates the impact of specific parameters or individual differences. In a randomized crossover trial (*N* = 42), university students practiced three 5-min, personalized slow-paced breathing protocols at 40%, 60%, and 80% of their spontaneous breathing frequency. Lower frequencies enhanced subjective relaxation and reduced physiological arousal, as indicated by decreased heart rate, increased heart rate variability, and increased peripheral skin temperature. Lower frequencies also induced a greater shift in prefrontal neurophysiological arousal, with decreased relative delta power and increased relative alpha and beta power. In addition, prior experience with relaxation techniques strengthened subjective benefits, whereas higher anxious-depressive symptoms were associated with greater perceived stress after slow-paced breathing. Personalization and parameter-based optimization of breathing protocols may enhance relaxation during practice and strengthen self-regulatory capacity.

## Highlights

• Breathing frequency shapes the relaxation response to paced breathing
• Slower breathing increases relaxation and shifts physiology toward lower arousal
• Slower breathing shifts prefrontal brain rhythms: delta down; alpha and beta up
• Responses vary with relaxation experience and anxious-depressive symptom severity

## Graphical abstract

**Figure:**
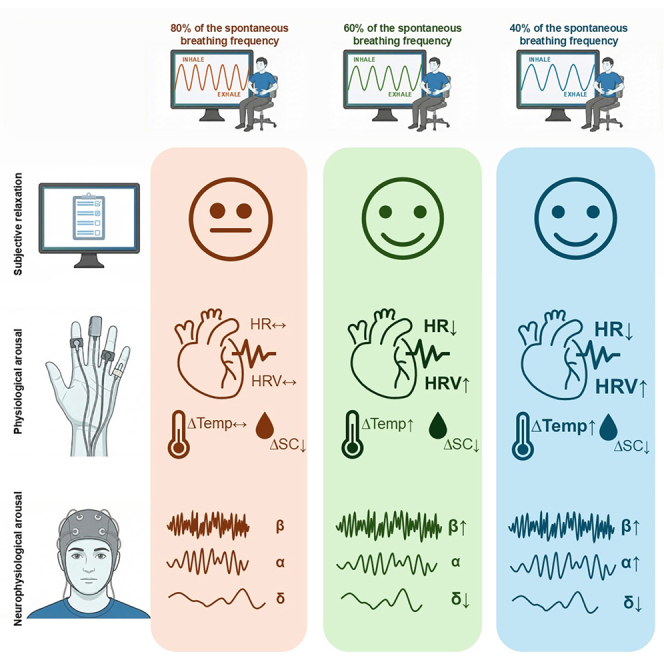

## Introduction

Arousal regulation is fundamental to human health. Within hours (ultradian rhythms) and throughout the day (circadian rhythms), we experience adaptive fluctuations in arousal.^1^^,^^2^ While arousal regulation allows us to cope with demands during the day and replenish resources at night, arousal dysregulation is detrimental to human health. Persistent demands that exceed an individual’s regulatory capacity and adjustive resources activate the human stress system and promote a chronic state of dysregulated hyperarousal.^3^^,^^4^^,^^5^^,^^6^ This hyperarousal disrupts executive functions, such as cognitive control and flexibility, favoring habitual over goal-directed behavior.^7^^,^^8^ It also compromises restorative functions, for example, contributing to impaired sleep initiation and maintenance^9^^,^^10^ as well as impaired digestion due to altered gut motility and permeability.^11^ Thus, persistent hyperarousal as a consequence of (chronic) stress increases the risk of many adverse health outcomes—ranging from cardiometabolic to mental health disorders.^12^

In general, humans have access to a vast set of actions to regulate arousal. We can go for a walk, talk to a friend, or, as common advice goes: take a few deep breaths. Notably, ancient Eastern traditions have long recognized the dual role of breathing as both a signal and a regulator of arousal.^13^^,^^14^ As the only vital function under both autonomic and volitional control, breathing provides a unique and ever-present lever to recognize and regulate arousal—making it a central component of almost all relaxation techniques.^15^ Overall, breathwork practices demonstrate (moderate) effectiveness in reducing stress and anxious-depressive symptoms; however, substantial variability in breathing protocols and their respective effectiveness presents a challenge.^16^^,^^17^

Addressing this challenge requires identifying key parameters and personal characteristics that enhance (or attenuate) the effectiveness of breathing protocols. In this randomized crossover trial, we investigate breathing frequency as a key parameter in slow-paced breathing protocols. We demonstrate that slower breathing, at approximately 40% or 60% (compared to 80%) of an individual’s spontaneous breathing frequency, yields greater self-reported relaxation, physiological de-arousal, and changes in prefrontal neurophysiological arousal. Furthermore, we show that individuals with greater relaxation technique expertise benefit (even) more from slower breathing frequencies, and we identify anxious-depressive symptom severity as associated with higher momentary perceived stress after slow-paced breathing. These findings contribute to the optimization and personalization of breathing protocols, and may pave the way for more effective, individualized arousal regulation strategies to promote stress resilience.

## Results

### A young, healthy participant cohort with substantial prior experience with relaxation techniques

**Figure 1:**
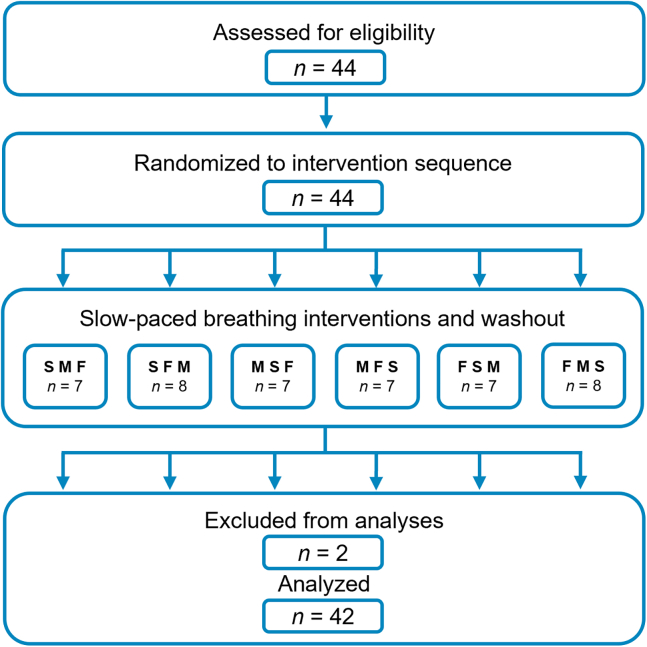
Participant flow and randomized crossover design of personalized slow-paced breathing protocols Participants practiced three 5-min slow (S), medium (M), and fast (F) slow-paced breathing protocols at 40%, 60%, and 80% of their individual spontaneous breathing frequency (measured at the beginning during either a period of distraction or rest, seated upright), respectively. Between breathing protocols, participants waited for 5 min to allow the effects of slow-paced breathing to wash out. We excluded two participants from the analyses because of an error in the pacer setting during one slow-paced breathing intervention, and one participant’s request to delete any personal data collected during the experiment.

We recruited participants through flyers, word of mouth, and advertisements through central channels of the Technical University of Munich from July 31 to November 27, 2023. We concluded the trial after reaching our estimated sample size of 42 participants, which required collecting data from 44 participants due to an error in the pacer setting during one breathing protocol and a request for data deletion by a single participant during the recruitment process. Figure 1 depicts participant flow through the study and the randomized crossover design. The experimental timeline and standardized slow-paced-breathing protocol parameters are provided in the STAR Methods.

Forty-four adult university students without any psychiatric, neurological, or cardiopulmonary disease diagnoses practiced three slow-paced breathing protocols, varying solely in the instructed breathing frequency, in a randomized sequence. Forty-two participants were included in all analyses. Table 1 summarizes participant characteristics, prior relaxation experience, and symptom levels.

**Table 1:** Participant characteristics, including prior relaxation experience and symptom levels

Female/male (total)	30/12 (42)
Mean age in years (SD)	23.6 (±2.7)
With/without experience with relaxation techniques^a^ (%)	34/8 (81/19)
Mean/median depressive symptoms^b^ (SD/IQR)	3.64/3 (±3.50/4)
Mean/median anxiety symptoms^b^ (SD/IQR)	3.31/3 (±2.49/3.75)
Mean/median stress symptoms^b^ (SD/IQR)	6/6 (±3.66/5)

Unless otherwise indicated, all values are based on the analyzed sample (*N* = 42).

a We assessed expertise with relaxation techniques with two questions on the frequency and duration of practice on a five-point Likert scale. Here, we report the number of participants who reported experience with relaxation techniques and those who reported none.

b We assessed depression, anxiety, and stress symptoms with the German version of the Depression-Anxiety-Stress-Scale by Nilges and Essau^19^—higher scores indicate greater symptom severity. Measures of central tendency are presented as the mean ± standard deviation (SD) for normal distribution, and in addition with the median with interquartile range (IQR) for skewed distribution.

### Participants adhered to the instructed breathing frequency during slow-paced breathing protocols and returned to spontaneous breathing between protocols

Paired-samples t-tests (Holm-adjusted for three comparisons) showed no significant differences between breathing frequency and instructed pacer frequency for all slow-paced breathing protocols. Additionally, a one-way repeated-measures analysis of variance showed no significant differences in practice duration across the three slow-paced breathing protocols. Table 2 reports the mean and standard deviation of pacer frequency, breathing frequency, and duration in seconds for each breathing protocol (slow, medium, and fast). It further provides the mean breathing frequency and duration, along with their standard deviations, for the distraction task, resting condition, and washout periods.

**Table 2:** Pacer frequency, breathing frequency, and duration across experimental conditions

	Condition						
	Distracted^a^	Resting^b^	Slow	Washout^c^	Medium	Washout^c^	Fast
Pacer frequency in breaths per minute	/	/	5.95 (±1.17)	/	8.91 (±1.79)	/	11.9 (±2.36)
Breathing frequency in breaths per minute	17.1 (±3.81)	11.2 (±3.26)	6.23 (±1.46)	14.1 (±3.58)	8.94 (±1.75)	14.5 (±3.00)	11.9 (±2.35)
Duration in seconds^d^	167 (±63.9)	306 (±2.91)	306 (±2.57)	325 (±19.9)	306 (±3.58)	331 (±42.3)	306 (±5.16)

Unless otherwise indicated, all values are based on the analyzed sample (*N* = 42). All data are presented as the mean ± standard deviation from the mean. Differences between breathing frequency and instructed pacer frequency for all breathing protocols (slow, medium, and fast) were tested by paired-samples t-tests (Holm-adjusted for three comparisons). Differences in the duration of breathing protocols were tested by one-way repeated-measures analysis of variance. No statistically significant differences were observed.

a During the distraction period, participants filled out a questionnaire and watched an instructional video.

b During the resting period, participants were instructed to sit still and fixate on a fixation cross displayed on a second monitor.

c During the washout, participants filled out a questionnaire and were asked to sit still.

d Small variations in the duration of the breathing protocols and washout periods resulted from minor inconsistencies in timekeeping by the principal investigator.

In 57% of participants (*n* = 24), the mean spontaneous breathing frequency was derived from the resting condition and used to determine the instructed breathing frequencies for the slow-paced breathing protocols (slow = 40%, medium = 60%, and fast = 80% of the spontaneous breathing frequency). In the remaining 43% of participants (*n* = 18), the breathing frequency during the resting condition fell outside the expected physiological range of 11–20 breaths per minute,^23^ so the distraction period was used instead. Figure S1 in the Supplemental Information (Document S1) demonstrates that the 5-min washout period was sufficient to minimize carry-over effects, with participants returning to spontaneous breathing and resting physiology.

### Lower breathing frequencies yield greater self-reported relaxation, physiological de-arousal, and changes in neurophysiological arousal

The theoretical effect model and linear mixed model structures are provided in the STAR Methods. Table 3 summarizes the linear mixed model results for self-report, physiological, and neurophysiological outcomes. We report the mean and confidence interval for each outcome and each breathing protocol. In addition, we report the estimated differences with 95% confidence intervals between slow-paced breathing protocols for all outcome measures using linear mixed models.

**Table 3:** Linear mixed model results for self-report, physiological, and neurophysiological outcomes

Outcomes	Mean (95% CI)			Slow vs. medium	Slow vs. fast	Medium vs. fast
	Slow	Medium	Fast	Estimate (95% CI)	Estimate (95% CI)	Estimate (95% CI)
**Self-report outcomes**						

RSQ	37.9 (34.7–41.0)	38.1 (34.9–41.3)	34.3 (31.1–37.5)	−0.238 (−2.85–2.37)	**3.57∗∗ (0.964–6.18)**	**3.81∗∗ (1.20–6.42)**
VAS	21.5 (14.4–28.7)	21.4 (14.3–28.6)	25.3 (18.2–32.4)	0.119 (−4.61–4.85)	−3.76 (−8.49–0.969)	−3.88 (−8.61–0.850)

**Physiological outcomes**						

HR	71.4 (67.0–75.7)	72.9 (68.6–77.3)	75.5 (71.1–79.9)	−1.57 (−3.60–0.456)	**−4.15 (−6.17–−2.12)∗∗∗**	**−2.58 (−4.60–−0.550)∗∗**
HRV	67.9 (58.1–77.7)	61.6 (51.8–71.3)	51.2 (41.5–61.0)	6.36 (−0.376–13.1)	**16.7∗∗∗ (9.96–23.4)**	**10.3∗∗ (3.60–17.1)**
Temp^a^^,^^b^	0.708 (0.356–1.06)	0.358 (0.006–0.710)	−0.005 (−0.358–0.347)	0.350 (−0.148–0.848)	**0.714∗∗ (0.216–1.21)**	0.363 (−0.135–0.861)
SC	−0.663 (−0.944–0.382)	−0.781 (−1.06–0.499)	−0.852 (−1.13–0.571)	0.118 (−0.084–0.320)	0.189 (−0.013–0.391)	0.071 (−0.131–0.273)

**Neurophysiological outcomes**						

Delta	40.9 (37.6–44.2)	41.5 (38.1–44.8)	43.1 (39.8–46.5)	−0.573 (−2.24–1.08)	**−2.24∗∗ (−3.89–−0.59)**	**−1.67∗ (−3.32–−0.020)**
Theta	19.1 (18.0–20.3)	19.0 (17.9–20.1)	19.4 (18.3–20.6)	0.119 (−0.493–0.732)	−0.309 (−0.922–0.304)	−0.428 (−1.04–0.184)
Alpha	15.3 (12.8–17.8)	14.8 (12.4–17.3)	14.4 (11.9–16.8)	0.485 (−0.278–1.25)	**0.946∗ (0.182–1.71)**	0.461 (−0.303–1.22)
Beta	16.1 (14.3–17.9)	16.1 (14.3–17.8)	15.0 (13.2–16.7)	0.032 (−0.876–0.940)	**1.15∗∗ (0.242–2.06)**	**1.12∗ (0.210–2.03)**
Gamma	4.90 (3.89–5.91)	4.94 (3.93–5.95)	4.52 (3.50–5.53)	−0.041 (−0.630–0.548)	0.386 (−0.202–0.975)	0.428 (−0.161–1.02)

Slow-paced breathing at 40% (slow), 60% (medium), and 80% (fast) of the spontaneous breathing frequency (measured at the beginning during a period of distraction or rest). RSQ = Mean sum score of the Relaxation State Questionnaire by Steghaus and Poth^18^ with ten items—higher scores indicate greater relaxation (range equals 10 to 50). VAS = Mean score of the visual analog scale value of momentary perceived stress—higher scores indicate greater stress (range equals zero to 100). HR = Mean of participant-level median heart rate in beats per minute. HRV = Root mean squared of successive differences (RMSSD) in milliseconds. Temp = Mean peripheral temperature change (difference score calculated by subtracting the mean of the first 5 s from the mean of the last 5 s for each breathing protocol) in degrees Celsius. SC = Mean skin conductance change (difference score calculated by subtracting the mean of the first 5 s from the mean of the last 5 s for each breathing protocol) in microsiemens. Mean relative power in percentages in the Delta (1–4 Hz), Theta (4–8 Hz), Alpha (8–13 Hz), Beta (13–30 Hz), and Gamma (30–45 Hz) frequency bands. Differences between slow, medium, and fast slow-paced breathing protocols (fixed effects) were analyzed using linear mixed models, controlling for random effects (participant ID and intervention sequence). P-values were Tukey-adjusted to control family-wise error across the three contrasts per outcome. Analyses were conducted using all participants with complete and valid data (*N* = 42).

a Room temperature was recorded and included as a covariate; it did not significantly predict peripheral temperature change (*p* = 0.147).

b Participant-level influence diagnostics according to Nieuwenhuis et al.^24^ (leave-one-participant-out Cook’s distance; threshold 4/n, *N* = 42) flagged no influential participants, and the slow versus fast contrast was unchanged (estimate = 0.714; Tukey-adjusted *p* = 0.003). Observation-level diagnostics according to Nieuwenhuis et al.^24^ (leave-one-observation-out Cook’s distance; threshold 4/n, *N* = 126) flagged 9 influential observations (slow: 5; medium: 2; fast: 2); excluding these attenuated the slow versus fast contrast (estimate = 0.297; 95% CI [−0.071, 0.666], Tukey-adjusted *p* = 0.138). Full details are provided in Table S2 in Document S1 of our Supplemental Information. Significant differences are displayed in bold. Significance is indicated with asterisks as follows: ∗*p* < 0.05, ∗∗*p* < 0.01, and ∗∗∗*p* < 0.001. CI = Confidence interval.

In addition, Table S1 and Figure S2 in the Supplemental Information (Document S1) provide an overview of the linear mixed model results, which are expanded to incorporate frequency-domain measures (low- and high-frequency) of heart rate variability and cover the resting condition. However, we excluded the resting condition as a baseline here because, for 43% of participants (*n* = 18), the spontaneous breathing frequency used to determine the breathing frequency during the slow-paced breathing protocols was derived from the distraction period rather than the resting condition. Figure S3 in our Supplemental Information makes this decision during the experiment transparent, and Table S3 provides sensitivity analyses demonstrating that the derivation from resting versus distraction during the experiment did not materially affect our results.

**Figure 2:**
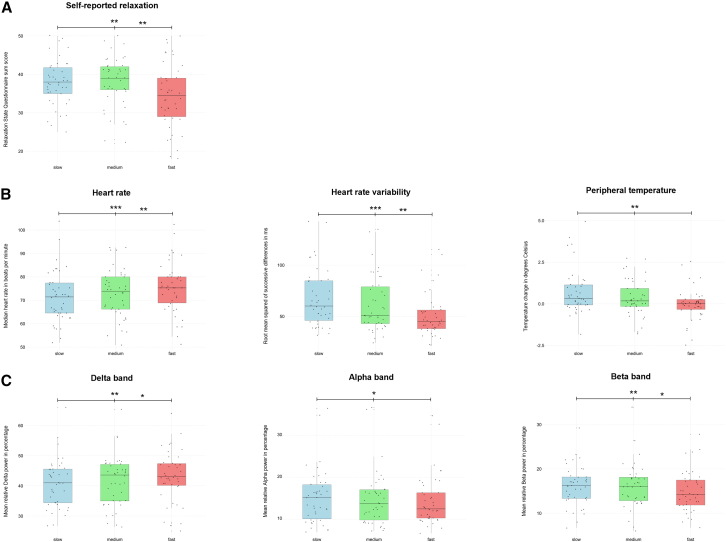
Lower breathing frequencies in slow-paced breathing yield greater self-reported relaxation, physiological de-arousal, and changes in neurophysiological arousal (A) Differences in Relaxation State Questionnaire sum scores by Steghaus and Poth^18^ (range 10–50, higher scores indicate greater relaxation) between slow-paced breathing protocols. (B) Differences in participant-level median heart rate in beats per minute, the root mean squared of successive differences (in heart rate) in milliseconds, and peripheral temperature (difference score calculated by subtracting the mean of the first 5 s from the mean of the last 5 s for each breathing protocol) in degrees Celsius between slow-paced breathing protocols. (C) Differences in mean relative power in percentages in the delta (1–4 Hz), alpha (8–13 Hz), and beta (13–30 Hz) frequency bands. Slow-paced breathing at 40% (slow, blue), 60% (medium, green), and 80% (fast, red) of the spontaneous breathing frequency (measured at the beginning, either during a period of distraction or rest, seated upright). Boxes represent the median and interquartile range (IQR), whiskers extend to the most extreme values within 1.5 times the IQR, and points represent individual observations. Differences between breathing protocols were tested using linear mixed models with Tukey-adjusted pairwise contrasts (*N* = 42). Significance is indicated with asterisks as follows: ∗*p* < 0.05, ∗∗*p* < 0.01, and ∗∗∗*p* < 0.001.

Figure 2 displays significant differences between slow-paced breathing protocols for self-report, physiological, and neurophysiological outcomes.

### Relaxation technique expertise moderates self-reported relaxation, and anxious-depressive symptom severity is associated with perceived stress after slow-paced breathing

There was an interaction effect between relaxation technique expertise and the slow-paced breathing protocol with the highest (versus lowest) breathing frequency on self-reported relaxation (estimate = −0.430, 95% CI [−0.800, −0.059], p = 0.028). In contrast, there were no interaction effects between slow-paced breathing protocols, self-reported relaxation, and symptoms of anxiety and depression. However, both anxiety and depression symptom severity were associated with momentary perceived stress after the slow-paced breathing protocols. Higher anxiety (estimate = 3.16, 95% CI [1.12, 5.12], *p* < 0.01) and depression symptom severity (estimate = 1.80, 95% CI [0.260, 3.25], *p* = 0.020) were both associated with increased momentary perceived stress.

**Figure 3:**
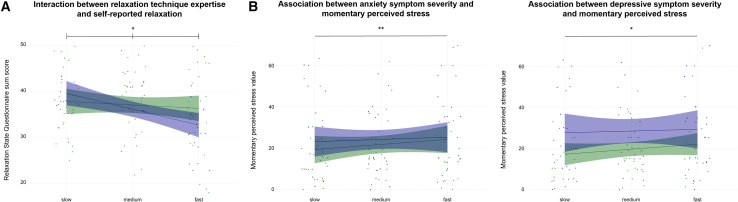
Relaxation technique expertise moderates self-reported relaxation, and anxious-depressive symptom severity is associated with perceived stress after slow-paced breathing (A) Interaction between relaxation technique expertise (index value as a product of the frequency and duration of practice values; range = 0–25, higher values indicate greater expertise) and self-reported relaxation (Relaxation State Questionnaire by Steghaus and Poth,^18^ range = 10–50, higher values indicate greater relaxation) after slow-paced breathing at 40% (slow), 60% (medium), and 80% (fast) of the spontaneous breathing frequency. (B) Associations of anxiety and depression symptom severity, assessed with the Depression-Anxiety-Stress Scale (DASS by Nilges and Essau,^19^ range = 0–21, respectively, higher values indicate greater symptom severity), with momentary perceived stress (rated on a visual analog scale, range = 0–100, higher values indicate greater momentary perceived stress) after slow-paced breathing at 40% (slow), 60% (medium), and 80% (fast) of the spontaneous breathing frequency. Significance was tested with continuous measures of relaxation technique expertise, anxiety symptoms, and depressive symptoms; however, to improve the visual discrimination of significant effects according to Allen and Erhardt,^25^ we divided our sample (*N* = 42) into approximately equal halves scoring low (green) or high (blue) in the respective characteristics. Points represent individual observations, solid lines represent fitted linear trends, and shaded bands represent 95% confidence intervals. Effects were tested using linear mixed model analyses. Significance is indicated with asterisks as follows: ∗*p* < 0.05 and ∗∗*p* < 0.01.

Figure 3 depicts the significant interaction between relaxation technique expertise and breathing protocols on self-reported relaxation, as well as the associations between anxious-depressive symptom severity and momentary perceived stress.

### Participants report a few minor adverse events after slow-paced breathing

Participants reported a few minor adverse events,^26^ including dizziness and shortness of breath, as well as minor discomfort, such as “uncomfortably long exhalation” or “stressful breathing without breathlessness.” Table 4 displays the adverse events and reported discomfort sorted by slow-paced breathing protocols.

**Table 4:** Minor adverse events and reported discomfort across slow-paced breathing protocols

	Slow	Medium	Fast
Dizziness	/	2	2
Shortness of breath	/	/	2
Other	1^a^	/	2^b^^,^^c^

Unless otherwise indicated, all values are based on the analyzed sample (*N* = 42).

a uncomfortably long exhalation.

b tension, cold hands, and some dizziness.

c stressful breathing without breathlessness.

## Discussion

This study demonstrates that breathing frequency is a key parameter in the effectiveness of slow-paced breathing protocols. A large-to-moderate reduction in breathing frequency to 40% or 60% of an individual’s spontaneous breathing frequency (approximately six or nine breaths per minute in our sample) yields greater subjective relaxation, physiological de-arousal, and changes in prefrontal neurophysiological arousal compared with faster slow-paced breathing at 80% of spontaneous breathing (approximately 12 breaths per minute in our sample).

We infer greater de-arousal during slow-paced breathing at lower breathing frequencies from decreases in heart rate, increases in heart rate variability, and increases in distal peripheral temperature; however, the temperature effect is sensitive to influential observations and should be interpreted cautiously. A decrease in heart rate alongside increased heart rate variability may indicate a shift in the autonomic balance of cardiac control.^27^^,^^28^ This shift may arise from a decrease in sympathetic tone, which accelerates heart rate, and/or an increase in parasympathetic tone, which decelerates heart rate and increases heart rate variability at rest.^27^^,^^29^ Although increased heart rate variability may largely reflect changes in breathing rather than cardiac parasympathetic tone,^30^ the concurrent decrease in heart rate in a resting state of 60–75 beats per minute supports a parasympathetic contribution to the observed effects.^27^^,^^29^ An increase in peripheral skin temperature may indicate increased cutaneous blood flow as a consequence of passive vasodilatation due to decreased sympathetic tone on digital blood vessels.^31^ In addition, a general decrease in peripheral skin conductance may indicate decreased sympathetic (sudomotor) tone on eccrine sweat glands across all slow-paced breathing protocols, albeit without significant differences between them.^32^^,^^33^

From the shift in prefrontal neurophysiological arousal, decreased relative power in the delta toward increased relative power in the alpha and beta frequency bands, we infer a state of relaxed alertness during slow-paced breathing at lower breathing frequencies. Elevated relative power in the alpha frequency band in prefrontal brain regions indicates cortical de-arousal,^34^^,^^35^ while elevated relative power in the beta frequency band may reflect heightened alertness and cognitive control maintained at lower breathing frequencies.^36^ However, the precise role of changes in power in the beta frequency band, particularly concerning specific brain regions, such as the prefrontal cortex, warrants further empirical investigation.^36^

Beyond the main effects, our results show interindividual differences in response to slow-paced breathing protocols. Participants with greater expertise in relaxation techniques report greater relaxation after slow-paced breathing at lower breathing frequencies, whereas participants with higher anxious-depressive symptom severity generally show higher momentary perceived stress after slow-paced breathing. These findings emphasize the need for a more personalized approach to breathwork practices, such as slow-paced breathing. Recent evidence similarly suggests that breath-based interventions show robust physiological effects, but more variable psychological effects, with substantial interindividual heterogeneity.^17^^,^^37^^,^^38^ In this context, prior experience may enhance benefit through better engagement and tolerability, consistent with prior work emphasizing the role of adherence and protocol engagement in breathing-based interventions,^39^ whereas higher symptom burden may be associated with altered response profiles, including greater residual stress in some individuals.^38^ These findings suggest that the optimization of breathwork may require personalization not only of breathing frequency, but also of protocol fit to individual characteristics and tolerability.

In conclusion, personalized slow-paced breathing at lower frequencies, compared with higher frequencies, enhances subjective relaxation and reduces physiological arousal. Prior experience with relaxation techniques appears to amplify subjective benefits, highlighting that breathing frequency and individual differences matter for optimizing breathing protocols. A key next step for breathwork research is to improve the mechanistic understanding of how specific parameters and individual characteristics shape the psychophysiological relaxation response, enabling more targeted recommendations. Such optimization may help individuals further strengthen self-regulatory capacity and support stress resilience by improving relaxation during breathwork practices.

### Limitations of the study

While this study provides important insights into the role of breathing frequency in slow-paced breathing protocols and individual differences in response to them, we want to highlight limitations and recommendations for future research.

First, we excluded the resting condition as a baseline in our mixed model analyses because 43% of participants had breathing frequencies outside the expected range of 11–20 breaths per minute during this period.^23^ Thus, in these cases, we estimated the spontaneous breathing frequency from the distraction period at the beginning of the experiment. As a result, the spontaneous breathing frequency used to derive the slow-paced breathing protocols may inaccurately reflect participants’ true spontaneous breathing. We recommend that future studies assess the spontaneous breathing frequency during a prolonged distraction task (e.g., reading or watching neutral content) or, preferably, during a full night of sleep, when autonomic breathing predominates.

Second, the majority (81%) of our participants reported prior experience with relaxation techniques, which is not representative of the general student population, in which less than half of all students practice relaxation techniques (28%); however, this number has almost doubled since 2015, highlighting increased interest in and adoption of relaxation techniques.^40^ This sampling bias may have resulted from self-selection, as participants received no monetary or study credit-related incentives. Future studies should employ broader recruitment strategies to improve sample heterogeneity and generalizability.

Third, we report nine minor adverse events, none of which led to dropout. This rate is unexpectedly high; a recent meta-analysis found that only one of the 12 studies reported such events.^17^ Notably, six of the nine adverse events were reported in regard to slow-paced breathing at 80% of the spontaneous breathing frequency. As breathing frequency covaries with other parameters, such as the 1:2 inhalation-to-exhalation ratio, it is unclear what exactly contributed to the reported discomfort. In general, we recommend a systematic assessment using the Common Terminology Criteria for Adverse Events (CTCAE)^26^ to better estimate the prevalence and types of adverse events, and thereby improve tolerability.

## Resource availability

### Lead contact

Further information and requests for resources should be directed to and will be fulfilled by the lead contact, Lukas Moebus (lukas.moebus@tum.de).

### Materials availability

This study did not generate new unique reagents.

### Data and code availability

• De-identified raw human physiology and survey data have been deposited on the Open Science Framework platform (https://doi.org/10.17605/OSF.IO/YQU9D). They are publicly available as of the date of publication.
• All original code has been deposited on the Open Science Framework platform (https://doi.org/10.17605/OSF.IO/YQU9D) and is publicly available as of the date of publication.
• Any additional information required to reanalyze the data reported in this paper is available from the lead contact upon request.

## Acknowledgments

We want to thank Philipp Gulde for reviewing an early draft of this manuscript.

## Author contributions

Conceptualization, L.M., F.E., and M.S.; methodology, L.M.; investigation, L.M.; writing – original draft, L.M.; writing – review and editing, F.E.; supervision, F.E. and M.S.

## Declaration of interests

The authors declare no competing interests.

## Declaration of generative AI and AI-assisted technologies in the writing process

During the preparation of this work, the authors used GitHub Copilot by Microsoft Corporation and Grammarly by Grammarly, Inc. to prepare and analyze the data as well as revise parts of the manuscript, respectively. After using these tools or services, the authors reviewed and edited the content as needed and take full responsibility for the content of the publication.

## STAR★Methods

### Key resources table

**Table:** 

REAGENT or RESOURCE	SOURCE	IDENTIFIER
**Deposited data**		

Raw and analyzed physiology and survey data	This paper; Open Science Framework	https://doi.org/10.17605/OSF.IO/YQU9D

**Software and algorithms**		

Original analysis code	This paper; Open Science Framework	https://doi.org/10.17605/OSF.IO/YQU9D
R (Version 4.4.0)	R Foundation	https://www.r-project.org/
BioTrace+ (Version V2018A1)	Mind Media Group	https://mindmedia.com/
G∗Power 3.1	The G∗Power Team	https://www.psychologie.hhu.de/arbeitsgruppen/allgemeine-psychologie-und-arbeitspsychologie/gpower

**Other**		

NeXus-10 MKII	Mind Media Group	https://mindmedia.com/

### Experimental model and study participant details

#### Human participants

Forty-two university students were included in the analyses of this randomized crossover trial. Participants were adults fluent in German, with a mean age of 23.6 years (SD = 2.7). The sample consisted of 30 self-reported female and 12 self-reported male participants. We excluded individuals who reported any psychiatric, neurologic, or cardiopulmonary disease. We did not collect data on race, ethnicity, or ancestry. Participants were allocated to a randomized, counterbalanced sequence of the three breathing protocols using a list generated in R prior to the trial.^41^ The Ethics Committee of the Technical University of Munich approved the study (2023-210-S-SR), and we conducted it in accordance with the Declaration of Helsinki.^42^ All participants gave written informed consent before participation. In an exploratory analysis, we tested whether the effects of breathing protocols differed by gender; no interaction reached statistical significance for any outcome. However, the study was not designed or powered to detect gender-based differences, and the generalizability of the findings across gender groups remains limited.

### Method details

#### Trial design

This randomized crossover trial was preregistered at ClinicalTrials.gov (NCT06121596). We followed the “CONSORT 2010 statement: extension to randomized crossover trials” and chose a crossover design because it is less susceptible to confounding effects due to unidentified differences in participant characteristics when comparing the effects of three five-minute slow-paced breathing protocols.^43^ However, such a design is susceptible to carryover and period effects. Thus, we chose a sufficient washout period of five minutes in between breathing protocols based on previous studies,^44^^,^^45^ and counterbalanced the random allocation sequence.^43^ We did not make any important changes to our methods after trial commencement.

#### Sample size and eligibility criteria

A large effect on our primary outcome of general relaxation would be of interest to us. Assuming a large effect in the difference between breathing protocols on our primary outcome of self-reported relaxation,^20^^,^^46^^,^^47^ with a two-sided significance of 0.05, and a power of 0.95, we recruited a total of 42 participants.^48^^,^^49^ Participants were included in this study if they were university students, above the age of 18, and fluent in German. Participants were excluded if they reported any psychiatric, neurologic, or cardiopulmonary disease diagnosis.^50^ We included university students because of a recent representative report on student health in Germany that reported a steep rise in the prevalence of high stress levels (almost doubling from 2015 to 2023) in this demographic.^40^^,^^51^

#### Procedures and interventions

We collected data from Monday to Friday between 12:00 and 18:00 in a quiet, dimly lit laboratory of the Technical University of Munich located in Munich, Germany.^52^ The experiment, as a whole, took approximately 80 minutes for each participant. The figure below shows the experimental timeline of the data collection procedure.

**Figure:**
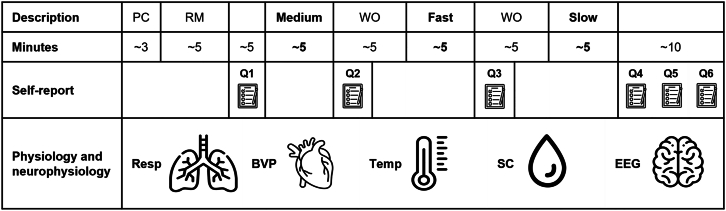
Experimental timeline of the data collection procedure PC = Plausibility check (distraction period) during which participants filled out a questionnaire (i.e., their adherence to the recommendations for data collection, such as not exercising vigorously or drinking alcohol the night before) and watched an instructional video on the resting measurement. RM = Resting measurement to assess spontaneous breathing frequency (measured during a period of distraction or rest, seated upright, looking at a fixation cross). Medium = Slow-paced breathing protocol at 60% of the spontaneous breathing frequency. WO = Washout. Fast = Slow-paced breathing protocol at 80% of the spontaneous breathing frequency. Slow = Slow-paced breathing protocol at 40% of the spontaneous breathing frequency. Q1 = Relaxation State Questionnaire by Steghaus and Poth^18^ with ten items (RSQ) and Visual Analog Scale of momentary perceived stress (VAS). Q2 = RSQ and VAS. Q3 = RSQ and VAS. Q4 = RSQ and VAS. Q5 = Adverse events, ease of practice, and expertise with relaxation techniques questionnaire. Q6 = Depression-Anxiety-Stress-Scale by Nilges and Essau^19^ with 21 items (DASS). Resp = Respiration frequency. BVP = Blood volume pulse. Temp = Skin temperature. SC = Skin conductance. EEG = Four-channel electroencephalography with linked-earlobe reference and active electrodes placed on F3, F4, F7, and F8.

Upon arrival at the laboratory, participants gave their written informed consent, after which we set up the experiment. During the plausibility check phase, participants provided demographic information such as age and gender, and confirmed their adherence to eligibility criteria and data collection guidelines. Additionally, they viewed a one-minute instructional video outlining the procedures for the resting measurement (RM). Subsequently, participants underwent the RM phase, during which they remained seated in an armchair and focused on a fixation cross displayed on a second monitor for five minutes. Following this, they completed the Relaxation State Questionnaire (RSQ), a ten-item questionnaire, and rated their momentary perceived stress level using a Visual Analog Scale (VAS) presented on the second monitor.

Participants then viewed a two-minute instructional video detailing the breathing protocols, were asked to address any remaining queries, and had the opportunity to practice the prescribed breathing technique (i.e., slow-paced abdominal, nasal breathing). Following this practice session, each participant practiced three personalized breathing protocols guided by a visual sinus wave pacer on the second monitor. The table below provides the parameters and settings of the breathing protocols. After completing each breathing protocol, participants again filled out the RSQ, rated their momentary stress level on the VAS, and waited for a five-minute interval to allow the effects to wash out before proceeding to the next protocol. We personalized the breathing frequency for each protocol based on the participant's spontaneous breathing frequency, which we defined as the mean breathing frequency during the RM. However, our pilot study indicated that some participants naturally regulate their breathing frequency (often lowering it) during the RM. To address this, we incorporated a plausibility check (PC)—a period in which participants were distracted, by filling out a questionnaire and watching an instructional video. If the mean breathing frequency during the RM fell outside the expected range of 11–20 breaths per minute,^23^ we calculated the spontaneous breathing frequency using the mean breathing frequency observed during the PC. Additionally, we bracketed the personalization in the aforementioned range to avoid hypo- and hyperventilation during breathing protocols. Consequently, participants practiced three personalized slow-paced breathing protocols in a randomized sequence. We allocated participants based on a randomized, counterbalanced allocation list generated prior to the trial with R programming.^41^

**Table:** Standardized parameters and settings of the slow-paced breathing protocols

Parameter^a^	Setting
Frequency^b^	40% (slow), 60% (medium), and 80% (fast) of the spontaneous breathing frequency
Rhythm^c^	inhalation-to-exhalation ratio of 1:2
Depth	deep into the abdomen
Quality^d^	nasal, “abdominal” breathing
Posture	seated with the spine erect in a comfortable and steady posture
Attention	attention on a visual pacer and the rising and falling of the abdomen
Framing^e^	in consent, paced breathing was introduced as a relaxation technique; during the breathing protocol, the pacer contained no text or labels, and participants were not told the target frequency or protocol type (slow, medium, or fast)—they were instructed solely to follow the pacer (but stop following the pacer if they feel any unease).

a In the design of our breathing protocol, we considered the checklist provided by Zaccaro et al.^16^

b Breathing frequency is the manipulated parameter that varies in its setting between breathing protocols.

c Breathing rhythm was set based on findings by Van Diest et al.^20^

d Nasal (vs. mouth) breathing was instructed as the preferred breathing mechanism based on findings by Zaccaro et al.^21^

e No deception or cover story was used based on findings by Szulczewski and Rynkiewicz.^22^

Upon completing all three breathing protocols, participants provided feedback on any adverse events they experienced during practice, the ease of practicing the breathing protocols, and their overall expertise with relaxation techniques. We assessed adverse events according to the Common Terminology Criteria for Adverse Events (CTCAE).^26^ Adverse events were assessed once at the end of the session, but participants reported them separately for each breathing protocol. If participants reported any discomfort during any breathing protocol, they were asked to select the breathing protocols and specify the discomfort as “Dizziness”, “Shortness of breath”, and/or “Other:”, which allowed them to describe their adverse event or discomfort in their own words. Additionally, they completed the Depression-Anxiety-Stress-Scale^19^ with 21 items.

#### Outcomes

We took a multimodal approach to assess relaxation and stress in response to breathing protocols. We included self-report, physiological, and neurophysiological outcomes. All physiological and neurophysiological outcomes were measured with the NeXus-10 MKII hardware and preprocessed using the corresponding latest BioTrace+ V2018 software (Mind Media B.V.; Herten, Netherlands). BioTrace+ was used to derive physiological time-series measures from the raw sensor signals. All subsequent processing, such as aggregating these software-derived time-series measures for each experimental condition and all statistical analyses, was performed in R.^41^

#### Self-report

Self-report outcomes include the RSQ^18^ with its ten items presented in random order and a VAS^53^ from zero (no stress) to 100 (absolutely stressed) of momentary perceived stress. The RSQ measures self-reported relaxation with ten items on a one-to-five-point Likert scale with a range from ten to 50. Higher scores indicate greater relaxation. Two exemplary items are “My muscles feel loose.” and “Right now, I am completely calm”. This questionnaire has good construct validity (examined through correlations with the Perceived Stress Questionnaire) and high reliability (Cronbach’s alpha = 0.86).^18^

#### Physiology

Physiological outcomes include blood volume pulse measured with a sampling rate of 128 Hz on the middle finger of the non-dominant hand to calculate the heart rate, the root mean square of successive differences in milliseconds (RMSSD),^54^ and the natural logarithm of low-frequency (0.04–0.15 Hz) and high-frequency (0.15–0.40 Hz) power (ln(ms^2^); which we report in Document S1 of our Supplemental Information) over each five-minute interval of slow-paced breathing. We chose RMSSD in our main analyses because it is a common measure of heart rate variability and is adequate for short-term measurements.^55^ Peripheral skin temperature with a sampling rate of 32 Hz was measured in degrees Celsius on the small finger of the non-dominant hand to calculate temperature changes. Skin conductance was measured in microsiemens with a sampling rate of 32 Hz on the index and ring fingers to calculate changes in skin conductance level. For skin temperature and conductance, we calculated a difference score in R by subtracting the mean value over a five-second time interval at the beginning from the mean value over a five-second time interval at the end of slow-paced breathing protocols because we expect a linear, relaxation-dependent increase and decrease in skin temperature and conductance, respectively.^32^ Sensitivity analyses using 10-, 30-, and 60-second averaging windows to calculate the difference score yielded comparable estimates, supporting the robustness of the five-second window choice. Full details are provided in Table S4 in Document S1 of our Supplemental Information. Additionally, breathing frequency was recorded at a sampling rate of 32 Hz using a respiratory belt to calculate the median breathing frequency over each five-minute slow-paced breathing interval from the derived respiration-rate time series to assess compliance with the instructed breathing frequency via the visual pacer.^56^ We selected the median due to its robustness against transient physiological artifacts (e.g., sighs or coughs) relative to the mean, and to avoid the mathematical instability of mode estimates for continuous high-precision values, which depend on binning/rounding choices.

#### Neurophysiology

The neurophysiological outcome includes EEG activity measured with a sampling rate of 512 Hz and bandpass filtered at 1–45 Hz. After applying abrasive gel (Nuprep, Weaver and Company) with a cotton swab on electrode sites of interest, we placed four active electrodes (silver/silver chloride discs) with conductive paste (Ten20, Weaver and Company) according to the International 10/20 System (F3, F4, F7, and F8) directly on the scalp. We chose the respective sites on the frontal lobe because of a meta-analysis on the EEG correlates of psychosocial stress that showed alpha power in frontal electrodes to inversely correlate with psychosocial stress.^35^ In addition, we placed reference electrodes on each earlobe for a linked earlobe referencing scheme and one ground reference electrode on the left mastoid.^57^ Movement artifacts were automatically detected and rejected by the BioTrace+ V2018 software using the built-in EMG artifact detection filter (75–100 Hz band).^58^ These artifact-free epochs were segmented into 2-second periods and extracted through a Hanning window with a 75% overlap. The Fast Fourier Transformation extracted relative power in percentages in the Delta- (1–4 Hz), Theta- (4–8 Hz), Alpha- (8–13 Hz), Beta- (13–30 Hz), and Gamma-band (30–45 Hz). We calculated mean relative power in the respective frequency bands over the entire prefrontal region (F3, F4, F7, F8) to identify changes in neurophysiological arousal.^59^^,^^60^

### Quantification and statistical analysis

#### Outcome analyses

We conducted linear mixed model (LMM) analyses for each outcome variable.^61^ Unless otherwise indicated, analyses were based on all participants with complete and valid data (*N* = 42), with repeated observations across breathing protocols modeled within participants. We used LMMs to test differences between breathing protocols for each outcome, including participant ID and intervention sequence as random intercepts. For peripheral temperature, room temperature was included as a covariate. Pairwise contrasts between breathing protocols were Tukey-adjusted. Statistical significance was defined as *p* < 0.05 using two-sided tests. Exact statistical details, including model estimates, confidence intervals, adjusted p values, and sample size, are reported in the results section and supplemental information. The analysis scripts, deposited data, and additional study materials are available on OSF.^62^ The figure below depicts the general structure of our expected effect model.^63^

**Figure:**
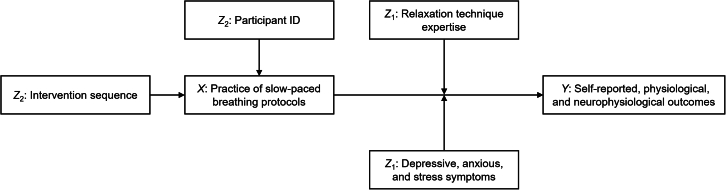
Theoretical effect model *X* = Fixed effects. *Y* = Outcomes. *Z*_2_ = Random effects. *Z*_1_ = Interaction effects. Intervention sequence is the randomly allocated, but overall counterbalanced sequence for each participant. Participant ID is the unique identifier of each participant. Slow-paced breathing at 40% (slow), 60% (medium), and 80% (fast) of the spontaneous breathing frequency (measured at the beginning during a period of distraction or rest, seated upright). Relaxation technique expertise is the index value as a product of the frequency and duration of practice values and ranges from zero (no expertise) to 25 (highest expertise). Depressive, anxious, and stress symptoms were measured with the Depression-Anxiety-Stress Scale by Nilges and Essau^19^ (range = 0–21 for each subscale).

#### Subgroup analyses

We conducted LMM analyses for our self-report outcomes, considering interaction effects between breathing protocols and participants' characteristics, such as expertise in relaxation techniques or anxious-depressive symptoms. The index value of relaxation technique expertise is a product of the duration and frequency value of self-reported practice in the past. Anxiety, depression, and stress symptom severity were operationalized as the sum scores of the respective subscales. To improve visual representation of the differences in self-reported relaxation in relation to participant characteristics shown in the main-text figure, we defined cut-offs to divide our sample into approximately two equal halves with no-to-low versus moderate-to-high expertise or symptom severity.^25^ The table below depicts the general structure of our LMMs, with and without interaction effects. As an exploratory analysis, we also fitted linear mixed models including a condition-by-gender interaction term for all outcomes.

**Table:** Linear mixed model structures

Outcomes (*Y*)	Self-report (RSQ and VAS) Physiology (HR, HRV, Temp, and SC) Neurophysiology (Relative power in the Delta, Theta, Alpha, Beta, and Gamma bands)
Fixed effects (*X*)	intervention (slow, medium, and fast slow-paced breathing)
Interaction effects (*Z*_*1*_)	relaxation technique expertise anxiety, depression, and stress symptom severity
Random effects (*Z*_*2*_)	intervention sequence (random intercept) participant ID (random intercept)

Model without interactions: Y ∼ *X* + *Z*_2_.

Model with interactions: Y ∼ *X* ∗*Z*_1_ + *Z*_2_.

RSQ = Mean sum score of the Relaxation State Questionnaire by Steghaus and Poth^18^ with ten items—higher scores indicate greater relaxation. VAS = Mean score of the visual analog scale value of momentary perceived stress—higher scores indicate more stress. HR = Mean of participant-level median heart rate in beats per minute. HRV = Root mean squared of successive differences (RMSSD) in milliseconds. Temp = Mean temperature change in degrees Celsius. SC = Mean skin conductance change in microsiemens. Mean relative power in percentages in the Delta (1–4 Hz), Theta (4–8 Hz), Alpha (8–13 Hz), Beta (13–30 Hz), and Gamma (30–45 Hz) frequency bands. Slow-paced breathing at 40% (slow), 60% (medium), and 80% (fast) of the spontaneous breathing frequency (measured during a period of distraction or rest, seated upright). Relaxation technique expertise is defined as a product of the duration and frequency values of self-reported practice in the past. Anxiety, depression, and stress symptom severity is the sum score for each subscale. Intervention sequence is a value between one and six, depending on the randomly allocated intervention sequence. Participant ID is the unique participant identifier. We used linear mixed models to test differences between breathing protocols for each outcome, including participant ID and intervention sequence as random intercepts. Pairwise contrasts between breathing protocols were Tukey-adjusted. Statistical significance was defined as *p* < 0.05 using two-sided tests. Analyses were conducted using all participants with complete and valid data (*N* = 42).

#### Adverse outcome reporting

We assessed the occurrence of adverse events according to the Common Terminology Criteria for Adverse Events (CTCAE).^26^ We report adverse events descriptively in tabular format.

### Additional resources

This study was preregistered at ClinicalTrials.gov (NCT06121596, https://clinicaltrials.gov/study/NCT06121596).

## References

[1] Hastings M.H., Maywood E.S., Brancaccio M. (2018). Generation of circadian rhythms in the suprachiasmatic nucleus. Nat. Rev. Neurosci..

[2] Lightman S.L., Birnie M.T., Conway-Campbell B.L. (2020). Dynamics of ACTH and Cortisol Secretion and Implications for Disease. Endocr. Rev..

[3] Agorastos A., Chrousos G.P. (2022). The neuroendocrinology of stress: the stress-related continuum of chronic disease development. Mol. Psychiatry.

[4] Lamotte G., Shouman K., Benarroch E.E. (2021). Stress and central autonomic network. Auton. Neurosci..

[5] Russell G., Lightman S. (2019). The human stress response. Nat. Rev. Endocrinol..

[6] Scott-Solomon E., Boehm E., Kuruvilla R. (2021). The sympathetic nervous system in development and disease. Nat. Rev. Neurosci..

[7] Arnsten A.F.T. (2015). Stress weakens prefrontal networks: molecular insults to higher cognition. Nat. Neurosci..

[8] Girotti M., Bulin S.E., Carreno F.R. (2024). Effects of chronic stress on cognitive function - From neurobiology to intervention. Neurobiol. Stress.

[9] Kalmbach D.A., Cuamatzi-Castelan A.S., Tonnu C.V., Tran K.M., Anderson J.R., Roth T., Drake C.L. (2018). Hyperarousal and sleep reactivity in insomnia: current insights. Nat. Sci. Sleep.

[10] Sulaman B.A., Wang S., Tyan J., Eban-Rothschild A. (2023). Neuro-orchestration of sleep and wakefulness. Nat. Neurosci..

[11] Kelly J.R., Kennedy P.J., Cryan J.F., Dinan T.G., Clarke G., Hyland N.P. (2015). Breaking down the barriers: the gut microbiome, intestinal permeability and stress-related psychiatric disorders. Front. Cell. Neurosci..

[12] O'Connor D.B., Thayer J.F., Vedhara K. (2021). Stress and Health: A Review of Psychobiological Processes. Annu. Rev. Psychol..

[13] Iyengar B.K.S. (2013).

[14] Rinpoche A., Zangmo A.C. (2013).

[15] Gerritsen R.J.S., Band G.P.H. (2018). Breath of Life: The Respiratory Vagal Stimulation Model of Contemplative Activity. Front. Hum. Neurosci..

[16] Zaccaro A., Piarulli A., Laurino M., Garbella E., Menicucci D., Neri B., Gemignani A. (2018). How Breath-Control Can Change Your Life: A Systematic Review on Psycho-Physiological Correlates of Slow Breathing. Front. Hum. Neurosci..

[17] Fincham G.W., Strauss C., Montero-Marin J., Cavanagh K. (2023). Effect of breathwork on stress and mental health: A meta-analysis of randomised-controlled trials. Sci. Rep..

[18] Steghaus S., Poth C.H. (2022). Assessing momentary relaxation using the Relaxation State Questionnaire (RSQ). Sci. Rep..

[19] Nilges P., Essau C. (2015). Depression, anxiety and stress scales. DASS—A screening procedure not only for pain patients. Schmerz.

[20] Van Diest I., Verstappen K., Aubert A.E., Widjaja D., Vansteenwegen D., Vlemincx E. (2014). Inhalation/Exhalation ratio modulates the effect of slow breathing on heart rate variability and relaxation. Appl. Psychophysiol. Biofeedback.

[21] Zaccaro A., Piarulli A., Melosini L., Menicucci D., Gemignani A. (2022). Neural Correlates of Non-ordinary States of Consciousness in Pranayama Practitioners: The Role of Slow Nasal Breathing. Front. Syst. Neurosci..

[22] Szulczewski M.T., Rynkiewicz A. (2018). The effects of breathing at a frequency of 0.1 Hz on affective state, the cardiovascular system, and adequacy of ventilation. Psychophysiology.

[23] Natarajan A., Su H.W., Heneghan C., Blunt L., O'Connor C., Niehaus L. (2021). Measurement of respiratory rate using wearable devices and applications to COVID-19 detection. npj Digit. Med..

[24] Nieuwenhuis R., Grotenhuis M., Pelzer B. (2012). influence.ME: Tools for Detecting Influential Data in Mixed Effects Models. The R Journal.

[25] Allen E.A., Erhardt E.B., Cacioppo J.T., Tassinary L.G., Berntson G.G. (2016). Handbook of Psychophysiology.

[26] National Cancer Institute. Common Terminology Criteria for Adverse Events (CTCAE). https://dctd.cancer.gov/research/ctep-trials/for-sites/adverse-events/ctcae-v5-5x7.pdf.

[27] Gordan R., Gwathmey J.K., Xie L.-H. (2015). Autonomic and endocrine control of cardiovascular function. World J. Cardiol..

[28] Wang T., Tufenkjian A., Ajijola O.A., Oka Y. (2025). Molecular and functional diversity of the autonomic nervous system. Nat. Rev. Neurosci..

[29] Karemaker J.M. (2022). The multibranched nerve: vagal function beyond heart rate variability. Biol. Psychol..

[30] Grossman P. (2024). Respiratory sinus arrhythmia (RSA), vagal tone and biobehavioral integration: Beyond parasympathetic function. Biol. Psychol..

[31] Tansey E.A., Johnson C.D. (2015). Recent advances in thermoregulation. Adv. Physiol. Educ..

[32] Dawson M.E., Schell A.M., Filion D.L., Cacioppo J.T., Tassinary L.G., Berntson G.G. (2016). Handbook of Psychophysiology.

[33] Ziemssen T., Siepmann T. (2019). The Investigation of the Cardiovascular and Sudomotor Autonomic Nervous System-A Review. Front. Neurol..

[34] Lomas T., Ivtzan I., Fu C.H.Y. (2015). A systematic review of the neurophysiology of mindfulness on EEG oscillations. Neurosci. Biobehav. Rev..

[35] Vanhollebeke G., De Smet S., De Raedt R., Baeken C., van Mierlo P., Vanderhasselt M.A. (2022). The neural correlates of psychosocial stress: A systematic review and meta-analysis of spectral analysis EEG studies. Neurobiol. Stress.

[36] Engel A.K., Fries P. (2010). Beta-band oscillations--signalling the status quo?. Curr. Opin. Neurobiol..

[37] Shao R., Man I.S.C., Lee T.M.C. (2024). The Effect of Slow-Paced Breathing on Cardiovascular and Emotion Functions: A Meta-Analysis and Systematic Review. Mindfulness.

[38] Vetter V.M., Kurth T., Konigorski S. (2026). Evaluation of two easy-to-implement digital breathing interventions in the context of daily stress levels in a series of N-of-1 trials: results from the Anti-Stress Intervention Among Physicians (ASIP) study. npj Digit. Med..

[39] Balban M.Y., Neri E., Kogon M.M., Weed L., Nouriani B., Jo B., Holl G., Zeitzer J.M., Spiegel D., Huberman A.D. (2023). Brief structured respiration practices enhance mood and reduce physiological arousal. Cell Rep. Med..

[40] Meyer B., Grobe T., Bessel S. (2023). Gesundheitsreport 2023 – Wie geht’s Deutschlands Studierenden?. https://www.tk.de/resource/blob/2146912/44b10e23720bf38c1559538949dd1078/gesundheitsreport-au-2023-data.pdf.

[41] R Core Team (2024).

[42] World Medical Association (2013). World Medical Association Declaration of Helsinki: Ethical Principles for Medical Research Involving Human Subjects. JAMA.

[43] Dwan K., Li T., Altman D.G., Elbourne D. (2019). CONSORT 2010 statement: extension to randomised crossover trials. BMJ.

[44] Russell M.E.B., Scott A.B., Boggero I.A., Carlson C.R. (2017). Inclusion of a rest period in diaphragmatic breathing increases high frequency heart rate variability: Implications for behavioral therapy. Psychophysiology.

[45] Gholamrezaei A., Van Diest I., Aziz Q., Vlaeyen J.W.S., Van Oudenhove L. (2019). Influence of inspiratory threshold load on cardiovascular responses to controlled breathing at 0.1 Hz. Psychophysiology.

[46] Schlatter S.T., Thérond C.C., Guillot A., Louisy S.P., Duclos A., Lehot J.J., Rimmelé T., Debarnot U.S., Lilot M.E. (2022). Effects of relaxing breathing paired with cardiac biofeedback on performance and relaxation during critical simulated situations: a prospective randomized controlled trial. BMC Med. Educ..

[47] Toussaint L., Nguyen Q.A., Roettger C., Dixon K., Offenbächer M., Kohls N., Hirsch J., Sirois F. (2021). Effectiveness of Progressive Muscle Relaxation, Deep Breathing, and Guided Imagery in Promoting Psychological and Physiological States of Relaxation. Evid. Based Complement. Alternat. Med..

[48] Noordzij M., Tripepi G., Dekker F.W., Zoccali C., Tanck M.W., Jager K.J. (2010). Sample size calculations: basic principles and common pitfalls. Nephrol. Dial. Transplant..

[49] Faul F., Erdfelder E., Buchner A., Lang A.G. (2009). Statistical power analyses using G∗Power 3.1: tests for correlation and regression analyses. Behav. Res. Methods.

[50] Laborde S., Allen M.S., Borges U., Hosang T.J., Furley P., Mosley E., Dosseville F. (2022). The Influence of Slow-Paced Breathing on Executive Function. J. Psychophysiol..

[51] Meyer B., Zill A., Dilba D. (2021). Entspann dich, Deutschland! TK-Stressstudie 2021. https://www.tk.de/resource/blob/2118106/cbdb7ed26363a35145d753516510f92d/stressstudie-2021-pdf-zum-download-data.pdf.

[52] Babiloni C., Barry R.J., Başar E., Blinowska K.J., Cichocki A., Drinkenburg W.H.I.M., Klimesch W., Knight R.T., Lopes da Silva F., Nunez P. (2020). International Federation of Clinical Neurophysiology (IFCN) - EEG research workgroup: Recommendations on frequency and topographic analysis of resting state EEG rhythms. Part 1: Applications in clinical research studies. Clin. Neurophysiol..

[53] Lesage F.X., Berjot S., Deschamps F. (2012). Clinical stress assessment using a visual analogue scale. Occup. Med..

[54] Task Force of the European Society of Cardiology the North American Society of Pacing Electrophysiology (1996). Heart rate variability: standards of measurement, physiological interpretation and clinical use. Task Force of the European Society of Cardiology and the North American Society of Pacing and Electrophysiology. Circulation.

[55] Shaffer F., Ginsberg J.P. (2017). An Overview of Heart Rate Variability Metrics and Norms. Front. Public Health.

[56] Lorig T.S., Cacioppo J.T., Tassinary L.G., Berntson G.G. (2016). Handbook of Psychophysiology.

[57] Mind Media B.V. (2018). Appendix 4: Linked-ear cable setup. EN - NeXus EEG User Guide V2.3. https://help.mindmedia.com/en_US/articles/421059-how-to-measure-eeg.

[58] Mind Media B.V. (2018). Analysis & Reporting. User Manual BioTrace+ Software. https://help.mindmedia.com/en_US/articles/421067-biotrace-software-user-manual.

[59] Kim D.-W., Im C.-H., Im C.-H. (2018). Computational EEG Analysis.

[60] Luck S.J., Kappenman E.S., Cacioppo J.T., Tassinary L.G., Berntson G.G. (2016). Handbook of Psychophysiology.

[61] Yu Z., Guindani M., Grieco S.F., Chen L., Holmes T.C., Xu X. (2022). Beyond t test and ANOVA: applications of mixed-effects models for more rigorous statistical analysis in neuroscience research. Neuron.

[62] Moebus L., Ehrlenspiel F., Spitschan M. (2025). Lower breathing frequencies in slow-paced breathing protocols enhance relaxation and reduce arousal: A randomized crossover trial.

[63] Pearl J. (2010). An Introduction to Causal Inference. Int. J. Biostat..

