# Supplementary material for: Lower breathing frequencies in personalized slow-paced breathing enhance relaxation and reduce arousal
Source: iScience. 2026 Apr 17;29(6):115803. doi: 10.1016/j.isci.2026.115803 (PMC13241781; doi:10.1016/j.isci.2026.115803)
Supplement: Document S1. Figures S1–S3 and Tables S1–S4 [file mmc1.pdf]

iScience, Volume *29*

Supplemental information

Lower breathing frequencies in personalized slow-paced breathing enhance relaxation and reduce arousal

Lukas Moebus, Manuel Spitschan, and Felix Ehrlenspiel

Figures S1, S2, and S3, Tables S1, S2, S3, and S4, and supplemental references

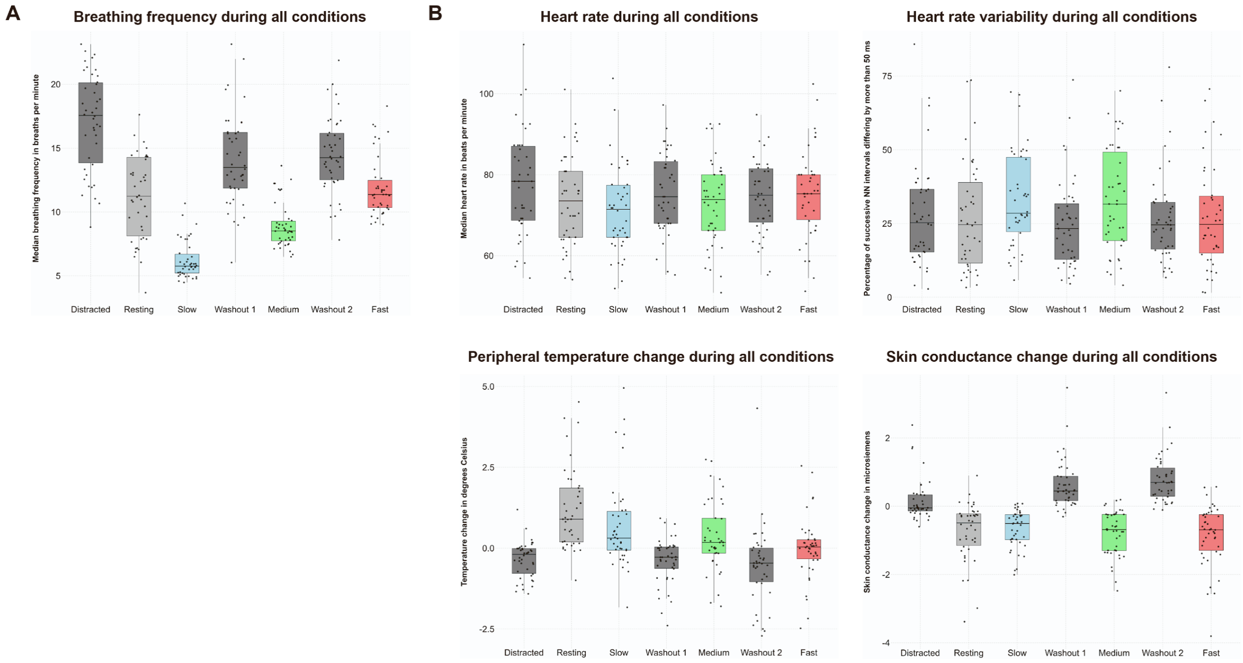

Figure S1. A five-minute washout period effectively minimizes carry-over effects in between slow-paced breathing protocols, Related to Table 2 and STAR Methods

(A) Participants returned to their spontaneous breathing frequency during the washout period. (B) Participants returned to a physiological resting state during five-minute washouts. Median heart rate in beats per minute increased. Heart rate variability, expressed as the percentage of successive normal-to-normal intervals differing by more than 50 ms (pNN50), decreased. We selected pNN50 here because it is more robust to artifacts (due to binary counting, pNN50 is less sensitive to single extreme values) than other short-term HRV indices. In contrast to our manuscript, we did not use the root mean square of successive differences (RMSSD) because artifacts were likely to occur during the washout periods, when some participants spoke or moved their hands while wearing the blood volume pulse sensor. Temperature and skin conductance changes (difference score calculated by subtracting the mean of the first five seconds from the mean of the last five seconds for each condition) show an increase and a decrease, respectively. We excluded one extreme value in this figure for the variable peripheral temperature change during the resting condition because of an implausibly high difference score of +9.05 degrees Celsius. This extreme value is unlikely to be a consequence of a relaxation response and was most likely an artifact due to a contact issue between the temperature sensor and the finger at the beginning of the resting condition.

Table S1. Overview of the linear mixed model results for self-report and physiological outcomes, including the resting condition, Related to Table 3 and Figure S2

Outcomes	Mean (95% CI)				Estimate (95% CI)					
	Resting	Slow	Medium	Fast	Resting vs. slow	Resting vs. medium	Resting vs. fast	Slow vs. medium	Slow vs. fast	Medium vs. fast
Self-report outcomes										
RSQ	35.3 (32.4–38.2)	37.9 (34.7–41.0)	38.1 (34.9–41.3)	34.3 (31.1–37.5)	-2.6 (-5.44–0.246)	-2.83 (-5.68–0.008)	0.976 (-1.87–3.82)	-0.238 (-2.85–2.37)	**3.57** (0.964–6.18)**	**3.81** (1.20–6.42)**
VAS	28.9 (22.1–35.7)	21.5 (14.4–28.7)	21.4 (14.3–28.6)	25.3 (18.2–32.4)	**7.36*** (2.54–12.1)**	**7.48*** (2.66–12.3)**	3.6 (-1.22–8.41)	0.119 (-4.61–4.85)	-3.76 (-8.49–0.969)	-3.88 (-8.61–0.850)
Physiological outcomes										
HR	73.5 (69.1–77.9)	71.4 (67–75.7)	72.9 (68.6–77.3)	75.5 (71.1–79.9)	**2.16* (0.135–4.19)**	0.591 (-1.43–2.62)	-1.98 (-4.01–0.041)	-1.57 (-3.6–0.456)	**-4.15*** (-6.17–-2.12)**	**-2.58** (-4.6–-0.55)**
RMSSD	54.7 (44.5–64.9)	67.9 (58.1–77.7)	61.6 (51.8–71.3)	51.2 (41.5–61.0)	**-13.2*** (-21.0–-5.47)**	-6.87 (-14.6–0.896)	3.47 (-4.29–11.2)	6.36 (-0.376–13.1)	**16.7*** (9.96–23.4)**	**10.3** (3.60–17.1)**
logLF	8.34 (7.93–8.74)	9.90 (9.50–10.3)	8.79 (8.39–9.20)	7.50 (7.09–7.91)	**-1.56*** (-1.99–-1.14)**	**-0.453* (-0.881–-0.024)**	**0.840*** (0.411–1.27)**	**1.11*** (0.683–1.54)**	**2.40*** (1.98–2.83)**	**1.29*** (0.864–1.72)**
logHF	7.99 (7.70–8.28)	7.77 (7.48–8.06)	7.75 (7.46–8.05)	8.32 (8.03–8.61)	0.217 (-0.208–0.642)	0.233 (-0.192–0.658)	-0.334 (-0.758–0.091)	0.016 (-0.409–0.441)	**-0.550** (-0.975–-0.125)**	**-0.566** (-0.991–-0.142)**

Temp	1.20 (0.841–1.57)	0.708 (0.349–1.07)	0.358 (-0.002–0.717)	-0.005 (-0.365–0.354)	0.496 (-0.053–1.05)	**0.846***** **(0.300–1.40)**	**1.21***** **(0.661–1.76)**	0.350 (-0.195–0.895)	**0.714**** **(0.169–1.26)**	0.363 (-0.182–0.908)
SC	-0.737 (-1.07–-0.409)	-0.663 (-0.991–-0.335)	-0.781 (-1.11–-0.453)	-0.852 (-1.18–-0.524)	-0.074 (-0.338–0.190)	0.044 (-0.220–0.308)	0.115 (-0.149–0.379)	0.118 (-0.146–0.382)	0.189 (-0.075–0.453)	0.071 (-0.193–0.335)

A five-minute resting condition (resting) seated upright, with eyes fixed on a fixation cross, and five-minute slow-paced breathing at 40% (slow), 60% (medium), and 80% (fast) of the spontaneous breathing frequency (measured at the beginning during a distraction or the resting period). RSQ = Mean sum score of the Relaxation State Questionnaire by Steghaus and Poth[S1] with ten items—higher scores indicate greater relaxation (range equals ten to 50). VAS = Mean score of the visual analog scale value of momentary perceived stress—higher scores indicate greater stress (range equals zero to 100). HR = Mean heart rate in beats per minute. RMSSD = Root mean squared of successive differences in milliseconds (ms). logLF = Natural logarithm of low-frequency (0.04–0.15 Hz) power ($\ln(ms^2)$). logHF = Natural logarithm of high-frequency (0.15–0.40 Hz) power ($\ln(ms^2)$). Temp = Mean temperature change (difference score calculated by subtracting the mean of the first five seconds from the mean of the last five seconds for each condition) in degrees Celsius. SC = Mean skin conductance change (difference score calculated by subtracting the mean of the first five seconds from the mean of the last five seconds for each condition) in microsiemens. Differences between resting, slow, medium, and fast slow-paced breathing protocols (fixed effects) were analyzed using linear mixed models controlling for random effects (participant ID and intervention sequence). Analyses were conducted using all participants with complete and valid data (N = 42). However, we excluded one extreme value for the variable Temp in the resting condition, as it had a difference score of +9.05 degrees Celsius, which is physiologically implausible. Significant differences are displayed in bold. Significance is indicated with asterisks as follows: *$p < 0.05$, **$p < 0.01$, ***$p < 0.001$. CI = Confidence interval.

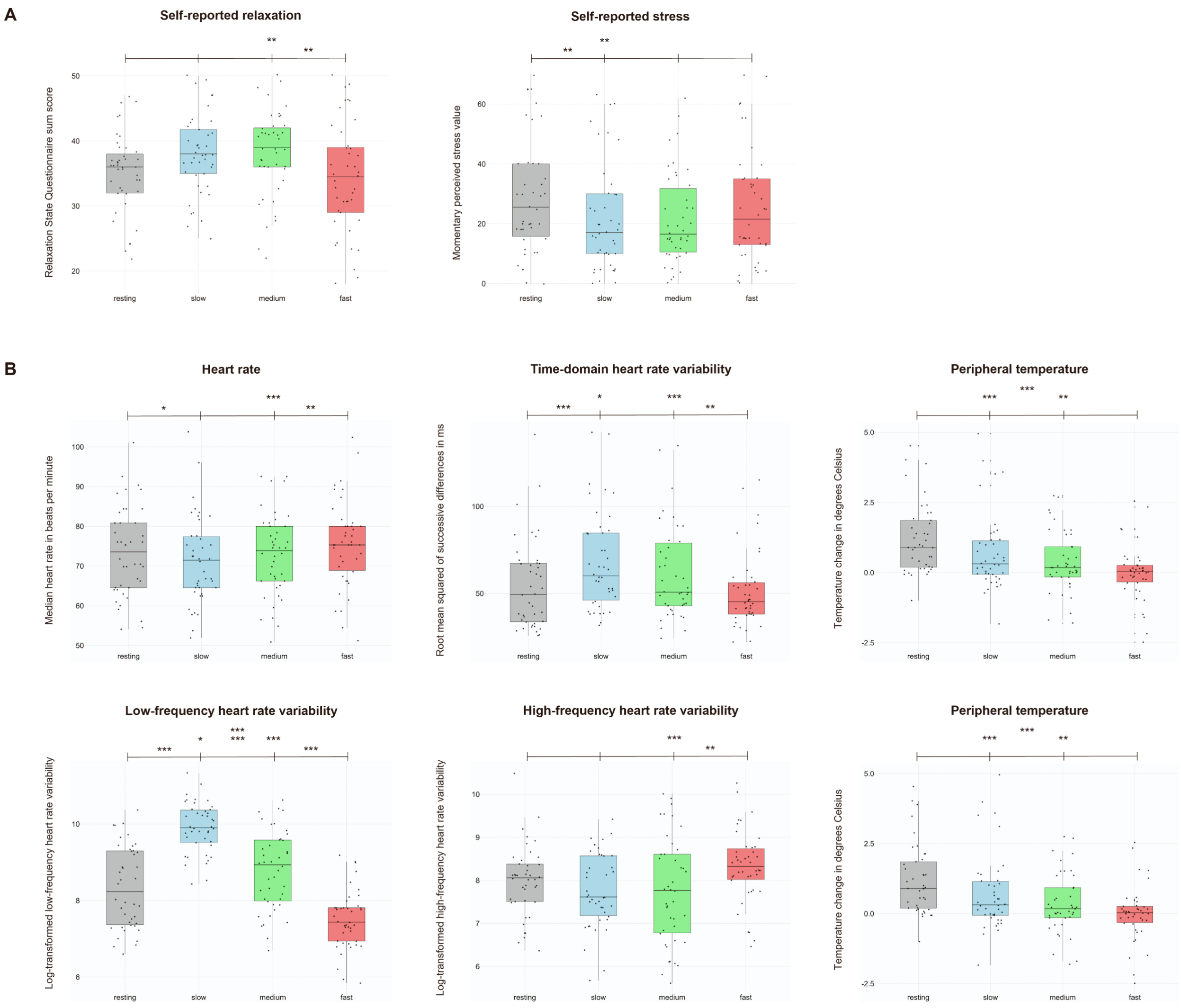

Figure S2. Slow-paced breathing compared to focused rest leads to higher subjective relaxation and lower momentary perceived stress, Related to Table 3 and Table S1

(A) Difference in Relaxation State Questionnaire sum scores by Steghaus and Poth[S1] (range ten to 50, higher scores indicate greater relaxation) and in the visual analog scale value of momentary perceived stress (range zero to 100, higher scores indicate greater stress) between rest and slow-paced breathing protocols. (B) Difference in median heart rate in beats per minute, in the root mean squared of successive differences (in heart rate) in milliseconds, in the natural logarithm of low-frequency (0.04–0.15 Hz) and high-frequency (0.15–0.40 Hz) power (ln[ms^2]), and in peripheral temperature change (difference score calculated by subtracting the mean of the first five seconds from the mean of the last five seconds for each condition) in degrees Celsius between rest and slow-paced breathing protocols. Resting (resting, grey) refers to a five-minute period of focused rest (participants looked at a fixation cross on a second monitor, seated upright) before the slow-paced breathing protocols. Slow-paced breathing at 40% (slow, blue), 60% (medium, green), and 80% (fast, red) of the spontaneous breathing frequency (measured at the beginning during a distraction or the resting period, seated upright). Significance is indicated with asterisks as follows: *$p < 0.05$, **$p < 0.01$, ***$p < 0.001$ by linear mixed model analyses.

Table S2. Sensitivity analyses of peripheral temperature change results, Related to Table 3

Analysis	Sample size (n)	Influence criterion	Contrast	Estimate	95% CI (lower, upper)	p-value (Tukey-adjusted)
Original (primary)	126		Slow vs. fast	0.714	[0.216, 1.21]	**0.003****
Participant-level influence removed (n=42; flagged=0)	126	Cook's D > 4/n; n = 42 participants	Slow vs. fast	0.714	[0.216, 1.21]	**0.003****
Observation-level influence removed (flagged=9)	117	Cook's D > 4/n; n = 126 observations	Slow vs. fast	0.297	[-0.071, 0.666]	0.138

All data are presented as linear mixed model estimates with 95% confidence intervals and Tukey-adjusted p-values for the family of three pairwise condition contrasts (slow, medium, and fast). Significance is indicated with asterisks as follows: **$p < 0.01$. Influential data points were identified using Cook's distance adapted for linear mixed models by Nieuwenhuis et al.[S2] Observation-level flagged observations were distributed across conditions (slow: 5, medium: 2, and fast: 2).

Overall, Table S2 shows that peripheral temperature change results were robust to participant-level influence (none of the participants exceeded the Cook's D threshold). In contrast, removing nine influential observations attenuated the slow versus fast contrast (estimate = 0.297; Tukey-adjusted p = 0.138), suggesting that this effect should be interpreted cautiously, as it depends on a small number of influential observations.

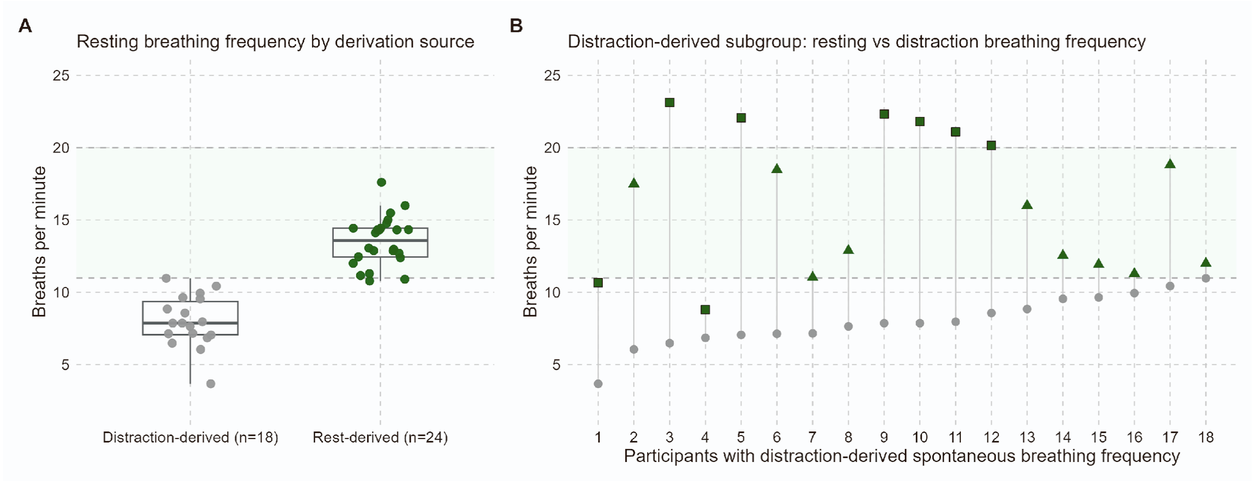

Figure S3. Transparency of spontaneous breathing frequency derivation for breathing protocol personalization, Related to STAR Methods and Table 3

The green-shaded band marks the physiological plausibility range of 11-20 breaths per minute (dashed lines), as reported by Natarajan et al.[S3] (A) Resting breathing frequency (dots) by derivation source. Participants whose spontaneous breathing frequency was rest-derived (n=24) had resting values within the plausibility range, whereas participants whose spontaneous breathing frequency was distraction-derived (n=18; including bracketing cases) showed resting values outside the range. (B) For the distraction-derived subgroup (n=18), paired values are shown for each participant (sorted by resting breathing frequency): resting breathing frequency (dots) and distraction-period breathing frequency (triangles and squares) used for personalization, with lines connecting within-participant pairs. Squares fell outside the expected physiological range and were bracketed to either 11 or 20 with the closest distance. This figure visualizes the rule-based, physiologically motivated decision (during the experiment) to derive the spontaneous breathing frequency from distraction when resting values were outside the expected range and therefore may lead to hyper- or hypoventilation.

Table S3. Sensitivity analyses with models adjusted for spontaneous breathing frequency derivation source, Related to Table 3

Outcomes	Slow vs. medium	Slow vs. fast	Medium vs. fast
	Estimate (95% CI)	Estimate (95% CI)	Estimate (95% CI)
Self-report outcomes			
RSQ	-0.238 (-2.85–2.37)	**3.57** (0.964–6.18)**	**3.81** (1.20–6.42)**
VAS	0.119 (-4.61–4.85)	-3.76 (-8.49–0.969)	-3.88 (-8.61–0.850)
Physiological outcomes			
HR	-1.57 (-3.60–0.456)	**-4.15 (-6.17–-2.12)*****	**-2.58 (-4.60–-0.550)****
HRV	6.36 (-0.376–13.1)	**16.7*** (9.96–23.4)**	**10.3** (3.60–17.1)**
Temp	0.350 (-0.148–0.848)	**0.714** (0.216–1.21)**	0.363 (-0.135–0.861)
SC	0.118 (-0.084–0.320)	0.189 (-0.013–0.391)	0.071 (-0.131–0.273)
Neurophysiological outcomes			
Delta	-0.573 (-2.24–1.08)	**-2.24** (-3.89–-0.59)**	**-1.67* (-3.32–-0.020)**
Theta	0.119 (-0.493–0.732)	-0.309 (-0.922–0.304)	-0.428 (-1.04–0.184)
Alpha	0.485 (-0.278–1.25)	**0.946* (0.182–1.71)**	0.461 (-0.303–1.22)
Beta	0.032 (-0.876–0.940)	**1.15** (0.242–2.06)**	**1.12* (0.210–2.03)**
Gamma	-0.041 (-0.630–0.548)	0.386 (-0.202–0.975)	0.428 (-0.161–1.02)

Slow-paced breathing at 40% (slow), 60% (medium), and 80% (fast) of the spontaneous breathing frequency (measured at the beginning during a period of distraction or rest). RSQ = Mean sum score of the Relaxation State Questionnaire by Steghaus and Poth[S1] with ten items—higher scores indicate greater relaxation (range equals ten to 50). VAS = Mean score of the visual analog scale value of momentary perceived stress—higher scores indicate greater stress (range equals zero to 100). HR = Mean of participant-level median heart rate in beats per minute. HRV = Root mean squared of successive differences (RMSSD) in milliseconds. Temp = Mean peripheral temperature change (difference score calculated by subtracting the mean of the first five seconds from the mean of the last five seconds for each breathing protocol) in degrees Celsius. SC = Mean skin conductance change (difference score calculated by subtracting the mean of the first five seconds from the mean of the last five seconds for each breathing protocol) in microsiemens. Mean relative power in percentages in the Delta (1–4 Hz), Theta (4–8 Hz), Alpha (8–13 Hz), Beta (13–30 Hz), and Gamma (30–45 Hz) frequency bands. Differences between slow, medium, and fast slow-paced breathing protocols (fixed effects) were analyzed using linear mixed models with spontaneous breathing frequency derivation (during resting versus distraction) as a covariate, controlling for random effects (participant ID and intervention sequence). P-values were Tukey-adjusted to control family-wise error across the three contrasts per outcome. Analyses were conducted using all participants with complete and valid data (N = 42). Significant differences are displayed in bold. Significance is indicated with asterisks as follows: *p < 0.05, **p < 0.01, ***p < 0.001. CI = Confidence interval.

Overall, linear mixed models for all Table 3 outcomes were adjusted for the derivation source of the spontaneous breathing frequency (used to calculate breathing frequencies during breathing protocols) as an additional fixed-effect covariate. Tukey-adjusted pairwise contrasts between the slow, medium, and fast breathing protocols remained unchanged relative to the primary models (max $|\Delta| < 0.001$).

Table S4. Sensitivity analyses of window length for peripheral temperature and skin conductance change results, Related to STAR Methods and Table 3

Analysis	Sample size (n)	Window length in seconds (s)	Contrast	Estimate	95% CI (lower, upper)	p-value (Tukey-adjusted)
Peripheral temperature change results						
Original (primary; 5s window)	126	5	Slow vs. medium	0.350	[-0.147, 0.848]	0.219
Original (primary; 5s window)	126	5	Slow vs. fast	0.714	[0.216, 1.21]	**0.003****
Original (primary; 5s window)	126	5	Medium vs. fast	0.363	[-0.135, 0.861]	0.196
Sensitivity (10s window)	126	10	Slow vs. medium	0.351	[-0.147, 0.848]	0.218
Sensitivity (10s window)	126	10	Slow vs. fast	0.713	[0.216, 1.21]	**0.003****
Sensitivity (10s window)	126	10	Medium vs. fast	0.362	[-0.135, 0.860]	0.197
Sensitivity (30s window)	126	30	Slow vs. medium	0.357	[-0.136, 0.851]	0.201
Sensitivity (30s window)	126	30	Slow vs. fast	0.716	[0.222, 1.21]	**0.002****
Sensitivity (30s window)	126	30	Medium vs. fast	0.358	[-0.136, 0.852]	0.200
Sensitivity (60s window)	126	60	Slow vs. medium	0.368	[-0.121, 0.857]	0.177
Sensitivity (60s window)	126	60	Slow vs. fast	0.712	[0.223, 1.20]	**0.002****
Sensitivity (60s window)	126	60	Medium vs. fast	0.344	[-0.145, 0.833]	0.219
Skin conductance change results						
Original (primary; 5s window)	126	5	Slow vs. medium	0.117	[-0.088, 0.322]	0.368
Original (primary; 5s window)	126	5	Slow vs. fast	0.189	[-0.017, 0.394]	0.078

Original (primary; 5s window)	126	5	Medium vs. fast	0.072	[-0.133, 0.277]	0.682
Sensitivity (10s window)	126	10	Slow vs. medium	0.105	[-0.101, 0.312]	0.447
Sensitivity (10s window)	126	10	Slow vs. fast	0.182	[-0.024, 0.389]	0.095
Sensitivity (10s window)	126	10	Medium vs. fast	0.077	[-0.130, 0.284]	0.649
Sensitivity (30s window)	126	30	Slow vs. medium	0.088	[-0.107, 0.283]	0.533
Sensitivity (30s window)	126	30	Slow vs. fast	0.158	[-0.037, 0.353]	0.136
Sensitivity (30s window)	126	30	Medium vs. fast	0.070	[-0.125, 0.265]	0.669
Sensitivity (60s window)	126	60	Slow vs. medium	0.079	[-0.107, 0.265]	0.572
Sensitivity (60s window)	126	60	Slow vs. fast	0.139	[-0.048, 0.325]	0.184
Sensitivity (60s window)	126	60	Medium vs. fast	0.060	[-0.127, 0.246]	0.725

All data are presented as linear mixed model estimates with 95% confidence intervals and Tukey-adjusted p-values for the family of three pairwise condition contrasts (slow, medium, and fast). For sensitivity analyses, peripheral temperature and skin conductance change scores were recalculated using the mean of the first and last 5, 10, 30, or 60 seconds of each five-minute interval. Significance is indicated with asterisks as follows: **$p < 0.01$.

Table S4 shows that window lengths (5, 10, 30, and 60 seconds) used to calculate peripheral temperature change values yielded stable results: estimates and confidence intervals were nearly identical, and the slow versus fast contrast remained significant (estimate ≈ 0.712–0.716; Tukey-adjusted $p \leq 0.003$). Skin conductance results were likewise stable: estimates changed only slightly with longer windows, and all contrasts remained non-significant after Tukey adjustment. Overall, the results remain effectively unchanged for varying window lengths to calculate the peripheral change values.

SUPPLEMENTAL REFERENCES

[S1] Steghaus, S., and Poth, C.H. (2022). Assessing momentary relaxation using the Relaxation State Questionnaire (RSQ). Sci Rep 12, 16341. 10.1038/s41598-022-20524-w.

[S2] Nieuwenhuis, R., Grotenhuis, M.t., and Pelzer, B. (2012). influence.ME: Tools for Detecting Influential Data in Mixed Effects Models. The R Journal 4, 38–47. 10.32614/RJ-2012-011.

[S3] Natarajan, A., Su, H.W., Heneghan, C., Blunt, L., O'Connor, C., and Niehaus, L. (2021). Measurement of respiratory rate using wearable devices and applications to COVID-19 detection. NPJ Digit Med 4, 136. 10.1038/s41746-021-00493-6.
